# (2*E*)-3-(3-Nitro­phen­yl)-1-[4-(piperidin-1-yl)phen­yl]prop-2-en-1-one

**DOI:** 10.1107/S1600536812008847

**Published:** 2012-03-07

**Authors:** Hoong-Kun Fun, Tze Shyang Chia, Prakash S. Nayak, B. Narayana, B. K. Sarojini

**Affiliations:** a X-ray Crystallography Unit, School of Physics, Universiti Sains Malaysia, 11800 USM, Penang, Malaysia; b Department of Studies in Chemistry, Mangalore University, Mangalagangotri 574 199, India; c Department of Chemistry, P. A. College of Engineering, Nadupadavu, Mangalore 574 153, India

## Abstract

In the title compound, C_20_H_20_N_2_O_3_, the piperidine ring adopts a chair conformation and its mean plane forms dihedral angles of 19.63 (9) and 19.44 (9)°, respectively, with the benzene and the nitro-substituted benzene ring. The benzene and nitro-substituted benzene rings are almost coplanar and make a dihedral angle of 4.78 (8)°. In the crystal, mol­ecules are linked by C—H⋯O hydrogen bonds into two-dimensional networks parallel to the *ab* plane. The crystal packing is further stabilized by π–π inter­actions [maximum centroid–centroid distance = 3.7807 (12) Å].

## Related literature

For related structures and background to chalcones, see: Fun *et al.* (2011*a* ▶,*b* ▶,*c* ▶,*d* ▶). For the stability of the temperature controller used in the data collection, see: Cosier & Glazer (1986 ▶). For ring conformations and ring puckering analysis, see: Cremer & Pople (1975 ▶). For reference bond lengths, see: Allen *et al.* (1987 ▶).
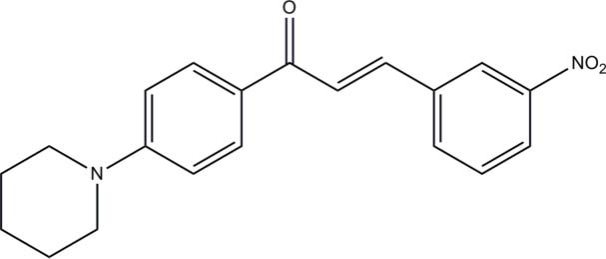


## Experimental

### 

#### Crystal data

C_20_H_20_N_2_O_3_
*M* *_r_* = 336.38
Orthorhombic, 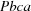

*a* = 7.4268 (12) Å
*b* = 11.3884 (18) Å
*c* = 39.447 (6) Å
*V* = 3336.4 (9) Å^3^
*Z* = 8
Mo *K*α radiation
μ = 0.09 mm^−1^
*T* = 100 K
0.30 × 0.22 × 0.11 mm

#### Data collection

Bruker APEX DUO CCD area-detector diffractometer
Absorption correction: multi-scan (*SADABS*; Bruker, 2009 ▶) *T*_min_ = 0.973, *T*_max_ = 0.990
20847 measured reflections
4870 independent reflections
3174 reflections with *I* > 2σ(*I*)
*R*_int_ = 0.062

#### Refinement

*R*[*F* ^2^ > 2σ(*F* ^2^)] = 0.056
*wR*(*F* ^2^) = 0.154
*S* = 1.04
4870 reflections
226 parameters
H-atom parameters constrained
Δρ_max_ = 0.29 e Å^−3^
Δρ_min_ = −0.23 e Å^−3^

### 

Data collection: *APEX2* (Bruker, 2009 ▶); cell refinement: *SAINT* (Bruker, 2009 ▶); data reduction: *SAINT*; program(s) used to solve structure: *SHELXTL* (Sheldrick, 2008 ▶); program(s) used to refine structure: *SHELXTL*; molecular graphics: *SHELXTL*; software used to prepare material for publication: *SHELXTL* and *PLATON* (Spek, 2009 ▶).

## Supplementary Material

Crystal structure: contains datablock(s) global, I. DOI: 10.1107/S1600536812008847/ds2176sup1.cif

Structure factors: contains datablock(s) I. DOI: 10.1107/S1600536812008847/ds2176Isup2.hkl

Supplementary material file. DOI: 10.1107/S1600536812008847/ds2176Isup3.cml

Additional supplementary materials:  crystallographic information; 3D view; checkCIF report

## Figures and Tables

**Table 1:** Hydrogen-bond geometry (Å, °)

*D*—H⋯*A*	*D*—H	H⋯*A*	*D*⋯*A*	*D*—H⋯*A*
C7—H7*A*⋯O3^i^	0.93	2.55	3.441 (2)	161
C16—H16*A*⋯O1^ii^	0.93	2.45	3.358 (2)	164

Symmetry codes: (i) 
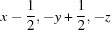
; (ii) 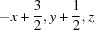.

**Fig. 1.**
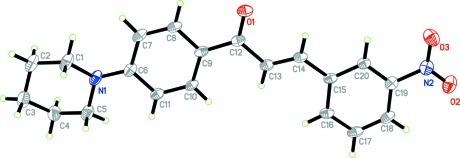
The molecular structure of the title compound with atom labels and 50% probability displacement ellipsoids.

**Fig. 2.**
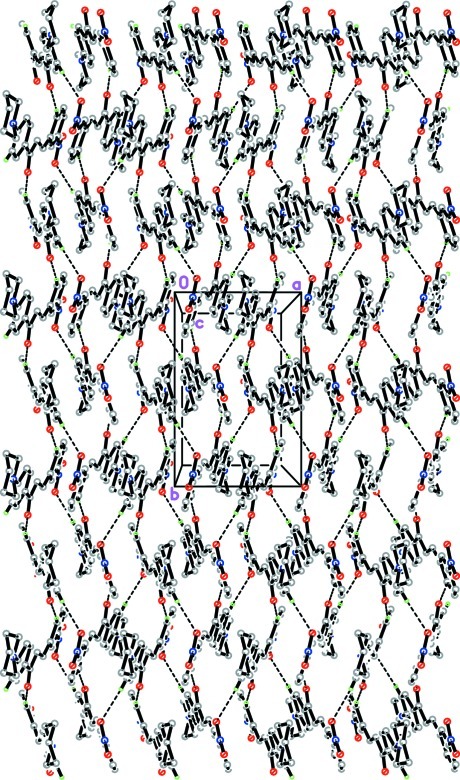
The crystal packing of the title compound. The dashed lines represent the hydrogen bonds. For clarity sake, hydrogen atoms not involved in hydrogen bonding have been omitted.

## supplementary crystallographic information

### Comment

In continuation of our work on synthesis of chalcones (Fun *et al.*, 2011*a*,*b*,*c*,*d*), the crystal structure of the title compound is reported here.

In the title compound (Fig. 1), the piperidine ring (N1/C1–C5) adopts a chair conformation [puckering parameters *Q* = 0.551 (2) Å, θ = 1.6 (2)° and φ = 233 (7)° (Cremer & Pople, 1975)] and form dihedral angles of 19.63 (9) and 19.44 (9)°, respectively with the benzene (C6–C11) and nitro-substituted benzene (C15–C20) ring. The essentially planar benzene [maximum deviation = 0.007 (1) Å at atoms C9 and C10] and nitro-substituted benzene ring [maximum deviation = 0.008 (2) Å at atom C17] are coplanar with each other, forming a dihedral angle of 4.78 (8)°. Bond lengths (Allen *et al.*, 1987) and angles are within normal ranges and are comparable to related structures (Fun *et al.*, 2011*a*,*b*,*c*,*d*).

In the crystal packing, the molecules are linked by intermolecular C—H···O hydrogen bonds into two-dimensional networks parallel to *ab* plane. The crystal packing is further stabilized by π–π interactions with *Cg*2···*Cg*3 = 3.7807 (12) and 3.7043 (12) Å (symmetry code = 1-*X*,1-Y,-*Z* and 2-*X*,1-Y,-*Z*, respectively), where *Cg*2 and *Cg*3 are the centroids of C6–C11 and C15–C20 rings respectively.

### Experimental

To a mixture of 4-piperidinoacetophenone (2.03 g, 0.01 mol) and 3-nitrobenzaldehyde (1.51 g, 0.01 mol) in ethanol (50 ml), 10 ml of 10% sodium hydroxide solution was added and stirred at 5–10 °C for 3 h. The precipitate formed was collected by filtration and purified by recrystallization from ethanol. The single-crystal was grown from mixture of acetone and toluene solvent by slow evaporation method (*M.P.*: 365–369 K).

### Refinement

All H atoms were positioned geometrically [C—H = 0.93 or 0.97 Å] and refined using a riding model with *U*_iso_(H) = 1.2 *U*_eq_(C).

### Crystal data

**Table:**

C_20_H_20_N_2_O_3_	*F*(000) = 1424
*M**_r_* = 336.38	*D*_x_ = 1.339 Mg m^−^^3^
Orthorhombic, *P**b**c**a*	Mo *K*α radiation, λ = 0.71073 Å
Hall symbol: -P 2ac 2ab	Cell parameters from 2665 reflections
*a* = 7.4268 (12) Å	θ = 3.1–29.6°
*b* = 11.3884 (18) Å	µ = 0.09 mm^−^^1^
*c* = 39.447 (6) Å	*T* = 100 K
*V* = 3336.4 (9) Å^3^	Block, orange
*Z* = 8	0.30 × 0.22 × 0.11 mm

### Data collection

**Table:**

Bruker APEX DUO CCD area-detector diffractometer	4870 independent reflections
Radiation source: fine-focus sealed tube	3174 reflections with *I* > 2σ(*I*)
Graphite monochromator	*R*_int_ = 0.062
φ and ω scans	θ_max_ = 30.0°, θ_min_ = 2.9°
Absorption correction: multi-scan (*SADABS*; Bruker, 2009)	*h* = −10→9
*T*_min_ = 0.973, *T*_max_ = 0.990	*k* = −16→16
20847 measured reflections	*l* = −55→47

### Refinement

**Table:**

Refinement on *F*^2^	Primary atom site location: structure-invariant direct methods
Least-squares matrix: full	Secondary atom site location: difference Fourier map
*R*[*F*^2^ > 2σ(*F*^2^)] = 0.056	Hydrogen site location: inferred from neighbouring sites
*wR*(*F*^2^) = 0.154	H-atom parameters constrained
*S* = 1.04	*w* = 1/[σ^2^(*F*_o_^2^) + (0.0603*P*)^2^ + 1.4691*P*] where *P* = (*F*_o_^2^ + 2*F*_c_^2^)/3
4870 reflections	(Δ/σ)_max_ < 0.001
226 parameters	Δρ_max_ = 0.29 e Å^−^^3^
0 restraints	Δρ_min_ = −0.23 e Å^−^^3^

### Special details

**Table:**

Experimental. The crystal was placed in the cold stream of an Oxford Cryosystems Cobra open-flow nitrogen cryostat (Cosier & Glazer, 1986) operating at 100.0 (1) K.
Geometry. All e.s.d.'s (except the e.s.d. in the dihedral angle between two l.s. planes) are estimated using the full covariance matrix. The cell e.s.d.'s are taken into account individually in the estimation of e.s.d.'s in distances, angles and torsion angles; correlations between e.s.d.'s in cell parameters are only used when they are defined by crystal symmetry. An approximate (isotropic) treatment of cell e.s.d.'s is used for estimating e.s.d.'s involving l.s. planes.
Refinement. Refinement of *F*^2^ against ALL reflections. The weighted *R*-factor *wR* and goodness of fit *S* are based on *F*^2^, conventional *R*-factors *R* are based on *F*, with *F* set to zero for negative *F*^2^. The threshold expression of *F*^2^ > σ(*F*^2^) is used only for calculating *R*-factors(gt) *etc*. and is not relevant to the choice of reflections for refinement. *R*-factors based on *F*^2^ are statistically about twice as large as those based on *F*, and *R*- factors based on ALL data will be even larger.

### Fractional atomic coordinates and isotropic or equivalent isotropic displacement parameters (Å^2^)

**Table:**

	*x*	*y*	*z*	*U*_iso_*/*U*_eq_	
O1	0.6504 (2)	0.26696 (11)	−0.01933 (3)	0.0378 (4)	
O2	0.9010 (2)	0.54808 (13)	0.17630 (4)	0.0448 (4)	
O3	0.8344 (2)	0.37615 (12)	0.15668 (3)	0.0388 (4)	
N1	0.5842 (2)	0.49503 (12)	−0.16639 (4)	0.0282 (4)	
N2	0.8685 (2)	0.48100 (13)	0.15277 (4)	0.0297 (4)	
C1	0.6185 (3)	0.40300 (17)	−0.19185 (5)	0.0370 (5)	
H1A	0.5738	0.3285	−0.1834	0.044*	
H1B	0.7473	0.3954	−0.1953	0.044*	
C2	0.5292 (3)	0.43002 (18)	−0.22548 (5)	0.0403 (5)	
H2A	0.5638	0.3710	−0.2420	0.048*	
H2B	0.3995	0.4266	−0.2228	0.048*	
C3	0.5819 (3)	0.55016 (19)	−0.23851 (5)	0.0372 (5)	
H3A	0.5138	0.5681	−0.2588	0.045*	
H3B	0.7089	0.5507	−0.2443	0.045*	
C4	0.5445 (3)	0.64214 (17)	−0.21165 (5)	0.0352 (5)	
H4A	0.4157	0.6478	−0.2080	0.042*	
H4B	0.5870	0.7179	−0.2195	0.042*	
C5	0.6370 (3)	0.61159 (16)	−0.17848 (5)	0.0333 (5)	
H5A	0.7664	0.6138	−0.1817	0.040*	
H5B	0.6059	0.6698	−0.1615	0.040*	
C6	0.6084 (2)	0.46749 (14)	−0.13234 (4)	0.0234 (4)	
C7	0.5554 (3)	0.35640 (14)	−0.11964 (5)	0.0260 (4)	
H7A	0.5074	0.3008	−0.1344	0.031*	
C8	0.5738 (3)	0.32971 (14)	−0.08587 (4)	0.0242 (4)	
H8A	0.5381	0.2560	−0.0783	0.029*	
C9	0.6450 (2)	0.41027 (13)	−0.06242 (4)	0.0215 (4)	
C10	0.6947 (2)	0.52043 (13)	−0.07476 (4)	0.0211 (3)	
H10A	0.7399	0.5763	−0.0598	0.025*	
C11	0.6784 (2)	0.54852 (14)	−0.10881 (4)	0.0229 (4)	
H11A	0.7144	0.6223	−0.1163	0.027*	
C12	0.6717 (3)	0.37118 (14)	−0.02707 (4)	0.0249 (4)	
C13	0.7283 (3)	0.45833 (14)	−0.00107 (4)	0.0241 (4)	
H13A	0.7355	0.5375	−0.0067	0.029*	
C14	0.7688 (3)	0.42367 (14)	0.03032 (4)	0.0243 (4)	
H14A	0.7605	0.3437	0.0348	0.029*	
C15	0.8250 (2)	0.49959 (13)	0.05843 (4)	0.0213 (3)	
C16	0.8867 (2)	0.61466 (14)	0.05323 (5)	0.0236 (4)	
H16A	0.8917	0.6449	0.0313	0.028*	
C17	0.9403 (3)	0.68375 (14)	0.08030 (5)	0.0272 (4)	
H17A	0.9826	0.7594	0.0763	0.033*	
C18	0.9318 (3)	0.64173 (14)	0.11314 (5)	0.0266 (4)	
H18A	0.9652	0.6887	0.1314	0.032*	
C19	0.8721 (2)	0.52768 (15)	0.11814 (4)	0.0236 (4)	
C20	0.8195 (2)	0.45610 (14)	0.09165 (4)	0.0221 (4)	
H20A	0.7809	0.3798	0.0958	0.027*	

### Atomic displacement parameters (Å^2^)

**Table:**

	*U*^11^	*U*^22^	*U*^33^	*U*^12^	*U*^13^	*U*^23^
O1	0.0575 (11)	0.0194 (6)	0.0366 (7)	−0.0105 (6)	−0.0024 (7)	−0.0006 (5)
O2	0.0530 (11)	0.0507 (9)	0.0308 (7)	−0.0046 (8)	−0.0067 (7)	−0.0089 (6)
O3	0.0516 (10)	0.0305 (7)	0.0342 (7)	0.0097 (7)	0.0058 (7)	0.0024 (6)
N1	0.0357 (10)	0.0217 (7)	0.0273 (7)	0.0073 (7)	−0.0075 (7)	−0.0091 (6)
N2	0.0293 (9)	0.0296 (8)	0.0301 (8)	0.0053 (7)	−0.0011 (7)	−0.0036 (6)
C1	0.0449 (14)	0.0328 (10)	0.0334 (10)	0.0072 (9)	−0.0069 (9)	−0.0158 (8)
C2	0.0478 (15)	0.0418 (11)	0.0312 (10)	0.0023 (10)	−0.0072 (9)	−0.0146 (8)
C3	0.0303 (12)	0.0533 (13)	0.0280 (9)	−0.0016 (10)	−0.0009 (8)	−0.0081 (9)
C4	0.0414 (13)	0.0365 (10)	0.0279 (9)	0.0028 (9)	−0.0026 (9)	0.0000 (8)
C5	0.0452 (14)	0.0261 (9)	0.0287 (9)	0.0047 (9)	−0.0062 (9)	−0.0064 (7)
C6	0.0218 (9)	0.0198 (7)	0.0287 (8)	0.0071 (7)	−0.0053 (7)	−0.0067 (6)
C7	0.0234 (10)	0.0200 (8)	0.0346 (9)	0.0018 (7)	−0.0059 (7)	−0.0100 (7)
C8	0.0208 (10)	0.0158 (7)	0.0361 (9)	−0.0002 (6)	−0.0011 (7)	−0.0049 (6)
C9	0.0181 (9)	0.0161 (7)	0.0303 (8)	0.0012 (6)	0.0001 (7)	−0.0048 (6)
C10	0.0195 (9)	0.0148 (7)	0.0290 (8)	0.0010 (6)	−0.0017 (7)	−0.0060 (6)
C11	0.0232 (10)	0.0159 (7)	0.0295 (8)	0.0028 (7)	−0.0013 (7)	−0.0048 (6)
C12	0.0247 (10)	0.0200 (7)	0.0300 (9)	−0.0017 (7)	0.0005 (7)	−0.0037 (6)
C13	0.0262 (10)	0.0167 (7)	0.0295 (9)	0.0003 (7)	0.0000 (7)	−0.0030 (6)
C14	0.0260 (10)	0.0159 (7)	0.0309 (9)	−0.0017 (7)	0.0006 (7)	−0.0014 (6)
C15	0.0180 (9)	0.0150 (7)	0.0307 (8)	0.0012 (6)	−0.0007 (7)	−0.0024 (6)
C16	0.0202 (9)	0.0169 (7)	0.0337 (9)	0.0009 (7)	0.0007 (7)	−0.0002 (6)
C17	0.0224 (10)	0.0157 (7)	0.0435 (10)	−0.0008 (7)	−0.0006 (8)	−0.0043 (7)
C18	0.0210 (10)	0.0215 (8)	0.0373 (10)	0.0008 (7)	−0.0036 (7)	−0.0095 (7)
C19	0.0189 (9)	0.0234 (8)	0.0285 (8)	0.0052 (7)	−0.0011 (7)	−0.0028 (6)
C20	0.0209 (9)	0.0163 (7)	0.0291 (8)	0.0022 (6)	−0.0007 (7)	−0.0017 (6)

### Geometric parameters (Å, º)

**Table:**

O1—C12	1.236 (2)	C7—H7A	0.9300
O2—N2	1.226 (2)	C8—C9	1.406 (2)
O3—N2	1.230 (2)	C8—H8A	0.9300
N1—C6	1.391 (2)	C9—C10	1.395 (2)
N1—C5	1.464 (2)	C9—C12	1.477 (2)
N1—C1	1.474 (2)	C10—C11	1.386 (2)
N2—C19	1.466 (2)	C10—H10A	0.9300
C1—C2	1.515 (3)	C11—H11A	0.9300
C1—H1A	0.9700	C12—C13	1.488 (2)
C1—H1B	0.9700	C13—C14	1.334 (2)
C2—C3	1.513 (3)	C13—H13A	0.9300
C2—H2A	0.9700	C14—C15	1.467 (2)
C2—H2B	0.9700	C14—H14A	0.9300
C3—C4	1.516 (3)	C15—C20	1.401 (2)
C3—H3A	0.9700	C15—C16	1.403 (2)
C3—H3B	0.9700	C16—C17	1.385 (2)
C4—C5	1.518 (3)	C16—H16A	0.9300
C4—H4A	0.9700	C17—C18	1.382 (3)
C4—H4B	0.9700	C17—H17A	0.9300
C5—H5A	0.9700	C18—C19	1.387 (2)
C5—H5B	0.9700	C18—H18A	0.9300
C6—C11	1.408 (2)	C19—C20	1.382 (2)
C6—C7	1.417 (2)	C20—H20A	0.9300
C7—C8	1.373 (2)		
			
C6—N1—C5	118.97 (14)	C6—C7—H7A	119.6
C6—N1—C1	118.37 (14)	C7—C8—C9	122.09 (16)
C5—N1—C1	112.13 (15)	C7—C8—H8A	119.0
O2—N2—O3	123.40 (16)	C9—C8—H8A	119.0
O2—N2—C19	118.41 (15)	C10—C9—C8	117.18 (16)
O3—N2—C19	118.19 (14)	C10—C9—C12	124.39 (15)
N1—C1—C2	112.11 (16)	C8—C9—C12	118.34 (15)
N1—C1—H1A	109.2	C11—C10—C9	121.49 (15)
C2—C1—H1A	109.2	C11—C10—H10A	119.3
N1—C1—H1B	109.2	C9—C10—H10A	119.3
C2—C1—H1B	109.2	C10—C11—C6	121.31 (15)
H1A—C1—H1B	107.9	C10—C11—H11A	119.3
C3—C2—C1	111.60 (18)	C6—C11—H11A	119.3
C3—C2—H2A	109.3	O1—C12—C9	120.37 (15)
C1—C2—H2A	109.3	O1—C12—C13	120.44 (16)
C3—C2—H2B	109.3	C9—C12—C13	119.18 (14)
C1—C2—H2B	109.3	C14—C13—C12	120.41 (15)
H2A—C2—H2B	108.0	C14—C13—H13A	119.8
C2—C3—C4	109.88 (17)	C12—C13—H13A	119.8
C2—C3—H3A	109.7	C13—C14—C15	126.26 (15)
C4—C3—H3A	109.7	C13—C14—H14A	116.9
C2—C3—H3B	109.7	C15—C14—H14A	116.9
C4—C3—H3B	109.7	C20—C15—C16	118.44 (15)
H3A—C3—H3B	108.2	C20—C15—C14	119.36 (15)
C3—C4—C5	111.17 (17)	C16—C15—C14	122.20 (15)
C3—C4—H4A	109.4	C17—C16—C15	120.77 (16)
C5—C4—H4A	109.4	C17—C16—H16A	119.6
C3—C4—H4B	109.4	C15—C16—H16A	119.6
C5—C4—H4B	109.4	C18—C17—C16	120.84 (16)
H4A—C4—H4B	108.0	C18—C17—H17A	119.6
N1—C5—C4	111.55 (16)	C16—C17—H17A	119.6
N1—C5—H5A	109.3	C17—C18—C19	118.19 (16)
C4—C5—H5A	109.3	C17—C18—H18A	120.9
N1—C5—H5B	109.3	C19—C18—H18A	120.9
C4—C5—H5B	109.3	C20—C19—C18	122.36 (16)
H5A—C5—H5B	108.0	C20—C19—N2	119.06 (15)
N1—C6—C11	122.43 (16)	C18—C19—N2	118.56 (15)
N1—C6—C7	120.46 (15)	C19—C20—C15	119.38 (15)
C11—C6—C7	117.06 (15)	C19—C20—H20A	120.3
C8—C7—C6	120.87 (15)	C15—C20—H20A	120.3
C8—C7—H7A	119.6		
			
C6—N1—C1—C2	160.67 (19)	C8—C9—C12—O1	7.8 (3)
C5—N1—C1—C2	−54.9 (2)	C10—C9—C12—C13	10.0 (3)
N1—C1—C2—C3	54.1 (3)	C8—C9—C12—C13	−173.55 (17)
C1—C2—C3—C4	−53.8 (3)	O1—C12—C13—C14	4.7 (3)
C2—C3—C4—C5	54.8 (2)	C9—C12—C13—C14	−174.04 (18)
C6—N1—C5—C4	−159.97 (17)	C12—C13—C14—C15	−179.56 (17)
C1—N1—C5—C4	55.8 (2)	C13—C14—C15—C20	164.14 (19)
C3—C4—C5—N1	−56.2 (2)	C13—C14—C15—C16	−16.6 (3)
C5—N1—C6—C11	1.4 (3)	C20—C15—C16—C17	−0.2 (3)
C1—N1—C6—C11	143.41 (19)	C14—C15—C16—C17	−179.45 (17)
C5—N1—C6—C7	178.68 (17)	C15—C16—C17—C18	−1.0 (3)
C1—N1—C6—C7	−39.3 (3)	C16—C17—C18—C19	1.5 (3)
N1—C6—C7—C8	−178.05 (17)	C17—C18—C19—C20	−0.7 (3)
C11—C6—C7—C8	−0.6 (3)	C17—C18—C19—N2	177.91 (16)
C6—C7—C8—C9	0.1 (3)	O2—N2—C19—C20	−174.07 (18)
C7—C8—C9—C10	0.9 (3)	O3—N2—C19—C20	6.5 (3)
C7—C8—C9—C12	−175.81 (17)	O2—N2—C19—C18	7.2 (3)
C8—C9—C10—C11	−1.4 (3)	O3—N2—C19—C18	−172.18 (17)
C12—C9—C10—C11	175.06 (17)	C18—C19—C20—C15	−0.5 (3)
C9—C10—C11—C6	0.9 (3)	N2—C19—C20—C15	−179.10 (16)
N1—C6—C11—C10	177.47 (17)	C16—C15—C20—C19	0.9 (3)
C7—C6—C11—C10	0.1 (3)	C14—C15—C20—C19	−179.82 (17)
C10—C9—C12—O1	−168.65 (18)		

### Hydrogen-bond geometry (Å, º)

**Table:**

*D*—H···*A*	*D*—H	H···*A*	*D*···*A*	*D*—H···*A*
C7—H7*A*···O3^i^	0.93	2.55	3.441 (2)	161
C16—H16*A*···O1^ii^	0.93	2.45	3.358 (2)	164

Symmetry codes: (i) *x*−1/2, −*y*+1/2, −*z*; (ii) −*x*+3/2, *y*+1/2, *z*.
